# Mitochondria of the Yeasts *Saccharomyces cerevisiae* and *Kluyveromyces lactis* Contain Nuclear rDNA-Encoded Proteins

**DOI:** 10.1371/journal.pone.0016325

**Published:** 2011-01-25

**Authors:** Aurélie Galopier, Sylvie Hermann-Le Denmat

**Affiliations:** 1 Université Paris-Sud, Orsay, France; 2 Institut de Génétique et Microbiologie, CNRS-UMR 8621, Orsay, France; 3 Ecole Normale Supérieure, Paris, France; University of Minnesota, United States of America

## Abstract

In eukaryotes, the nuclear ribosomal DNA (rDNA) is the source of the structural 18S, 5.8S and 25S rRNAs. In hemiascomycetous yeasts, the 25S rDNA sequence was described to lodge an antisense open reading frame (ORF) named *TAR1* for Transcript Antisense to Ribosomal RNA. Here, we present the first immuno-detection and sub-cellular localization of the *authentic* product of this atypical yeast gene. Using specific antibodies against the predicted amino-acid sequence of the *Saccharomyces cerevisiae TAR1* product, we detected the endogenous Tar1p polypeptides in *S. cerevisiae* (*Sc*) and *Kluyveromyces lactis* (*K*l) species and found that both proteins localize to mitochondria. Protease and carbonate treatments of purified mitochondria further revealed that endogenous *Sc* Tar1p protein sub-localizes in the inner membrane in a N_in_-C_out_ topology. Plasmid-versions of 5′ end or 3′ end truncated *TAR1* ORF were used to demonstrate that neither the N-terminus nor the C-terminus of *Sc* Tar1p were required for its localization. Also, Tar1p is a presequence-less protein. Endogenous *Sc* Tar1p was found to be a low abundant protein, which is expressed in fermentable and non-fermentable growth conditions. Endogenous *Sc TAR1* transcripts were also found low abundant and consistently 5′ flanking regions of *TAR1* ORF exhibit modest promoter activity when assayed in a luciferase-reporter system. Using rapid amplification of cDNA ends (RACE) PCR, we also determined that endogenous *Sc TAR1* transcripts possess heterogeneous 5′ and 3′ ends probably reflecting the complex expression of a gene embedded in actively transcribed rDNA sequence. Altogether, our results definitively ascertain that the antisense yeast gene *TAR1* constitutes a functional transcription unit within the nuclear rDNA repeats.

## Introduction

**Figure 1:**
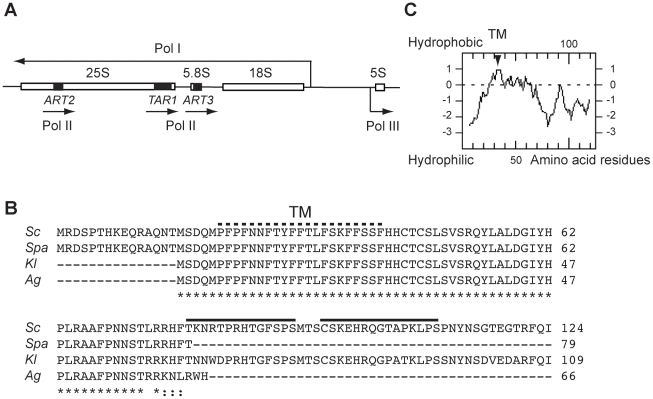
*TAR1* nested antisense gene and Tar1p protein sequence. (A) Diagram of one *S. cerevisiae* rDNA repeat unit showing the polymerase I (Pol I) transcript (processed into mature 18S, 5.8S, and 25S rRNA), the polymerase III (Pol III) transcribed gene 5S, and the polymerase II (Pol II) transcribed genes *TAR1* (375 bp), *ART2* (186 bp) and *ART3* (204 bp). Position of the Pol II genes within the rDNA sequence is represented by black boxes. Each arrow indicates the direction of transcription. (B) Protein sequence alignment of Tar1p from *S. cerevisiae* (*Sc*), *Saccharomyces paradoxus* (*Spa*), *Kluyveromyces lactis* (*Kl*) and *Ashbya gossypii* (*Ag*) species. Numbering refers to the entire predicted product of corresponding *TAR1* genes. Star indicates identity and dots similarity. Black lines indicate the C-terminal peptides of *Sc* Tar1p chosen for polyclonal antibodies production. Dotted line indicates the putative transmembrane domain (TM). (C) Hydropathy plot of *Sc* Tar1p [40]. The putative TM is indicated.

In *Saccharomyces cerevisiae* the ribosomal DNA (rDNA) locus is unique, located on chromosome XII and composed of 150 to 200 units repeated in tandem [1]. Each unit contains the 18S, 5.8S and 25S rRNA genes transcribed by RNA polymerase I (Pol I) as a unique 35S pre-rRNA and the 5S rRNA gene transcribed by RNA polymerase III (Pol III) (see Figure 1A). Whereas rDNA is highly transcribed by Pol I and III [2], Pol II-transcribed genes integrated into the rDNA units are silenced [3] [4] (and references therein). Despite the rDNA silencing of Pol II genes, chromatin immunoprecipitation (ChIP) analyses have revealed sites of yeast Pol II occupancy in the rDNA [5]. Additionally, coding-sequences nested in the rDNA have been trapped in an approach based on transposon tagging with a *lacZ* reporter that lacks both promoter sequences and an initiator ATG codon [6]. Insertions that produced protein fusions to β-galactosidase were thus identified in three small open reading frames (ORF) antisense to the rDNA. They were named *ART1* (hereafter *TAR1*), *ART2* and *ART3* [6]. Whereas *TAR1* (Transcript Antisense to Ribosomal RNA) and *ART2* are on the opposite strand of the 25S rDNA, *ART3* stands opposite to the 5.8S rDNA (Figure 1A). In *S. cerevisiae*, the *TAR1* ORF is 375 base pairs (bp) long and possesses a codon adaptation index (CAI) of 0.169 that is indicative of a sequence likely to be expressed [7]. All of the insertions of *lacZ* in the *TAR1* sequence were indeed reported to yield β-galactosidase activity [8]. In comparison, the *ART2* and *ART3* ORF are shorter (186 bp and 204 bp, respectively), possess a lower CAI index (0.086 and 0.105, respectively) and detailed expression of the *ART2-lacZ* and *ART3-lacZ* in frame-fusions was not reported.

In a genetic approach aimed to select for yeast factors interfering with mitochondrial import, we had isolated portions of a nuclear rDNA unit that included the *TAR1* and *ART3* ORF [9] (and unpublished data). Nevertheless, neither *TAR1* nor *ART3* were found involved in the improvement of the respiratory growth we observed in our strains (unpublished data). Selection of nuclear rDNA fragments acting as genetic suppressors was independently described in a screen that used a mutant of the Rpo41p mitochondrial RNA polymerase [8]. In this case, while a moderate expression of *TAR1* ORF was found to rescue the respiration-deficient phenotype of the *rpo41* mutant [8], a high expression exacerbated the defects of the mutant [10]. Genetic interaction between the rDNA-nested *TAR1* ORF and the *RPO41* gene is thus unclear as is the selection of nuclear rDNA portions in genetic screens based on the rescue of respiration-deficient phenotypes in yeast. Two decades ago, other links associating the respiratory-function of mitochondria and the nuclear rDNA locus had been reported. A differential expression of transcripts derived from the rDNA locus had thus been observed between respiratory competent and respiratory deficient yeast cells [11] [12]. In addition, it was found that respiratory deficient cells could show a tendency to trigger the polymerase switch from RNA Pol I to RNA Pol II in the synthesis of the rRNA [13]. So, amazing connections between yeast mitochondria and the nuclear rDNA locus exist but they stay poorly characterized and little studied.

In the present work, we establish for the first time that the rDNA-nested *TAR1* ORF of the yeasts *S. cerevisiae* (*Sc*) and *Kluyveromyces lactis* (*Kl*) codes for an authentic endogenous protein, which is specifically immuno-detected in the mitochondria of both species. Using a combination of biochemical and molecular approaches, we found that Tar1p is a presequence-less protein, which is anchored in the inner membrane by one transmembrane domain. Considering Tar1p amino-acid sequence, we propose that mitochondrial localization uses an internal amphipathic α-helix. We found that endogenous *Sc* Tar1p was similarly detectable in glucose and galactose medium while being less detectable under respiratory conditions. Finally, we present data underlining the complex transcriptional expression of the *TAR1* gene and discuss about the *cis-* and *trans*-elements that could regulate the expression of this atypical gene.

## Results

### In *S. cerevisiae* and *K. lactis*, the rDNA-nested *TAR1* gene codes for an authentic protein located in mitochondria

In *S. cerevisiae*, *TAR1* is predicted to encode a polypeptide of 124 amino acids (aas, Figure 1B). We obtained polyclonal antibodies raised against two C-terminal peptides of the *Sc* Tar1p amino-acid sequence that were predicted to be antigenic: TKNRTPRHTGFSPS (residues 79 to 92, residue 1 being the first methionine of *TAR1* ORF; Figure 1B) and CSKEHRQGTAPKLPS (residues 96 to 110). Antibodies affinity purified against the peptide TKNRTPRHTGFSPS (hereafter anti-Tar1p antibody) gave rise to a better immuno-detection and were used for the western blot analyses showed in this study.

**Figure 2:**
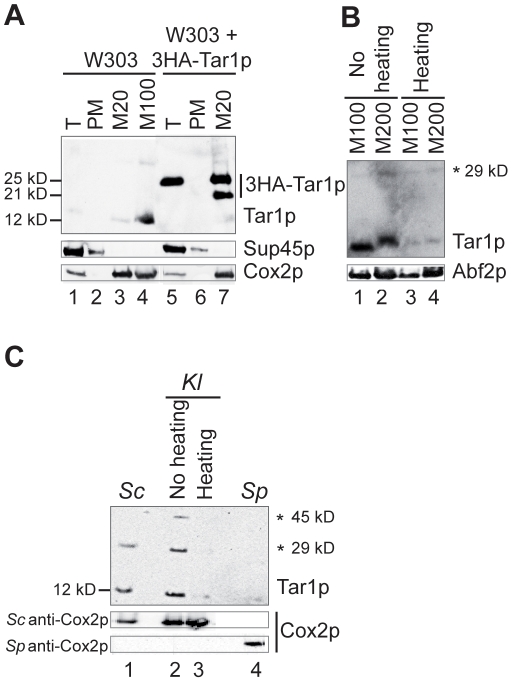
Endogenous Tar1p from *S. cerevisiae* (*Sc*) and *K. lactis* (*Kl*) species co-fractionate with mitochondria. Yeast cells were grown in galactose containing medium, disrupted to yield total cell extracts (T), and fractionated into mitochondrial pellets (M) and postmitochondrial supernatants (PM). Extracts were resolved on SDS-polyacrylamide gel electrophoresis and subjected to immunoblotting. Markers for the different subcellular fractions were Sup45p for cytosol, Cox2p and Abf2p for mitochondria. *Sc* Tar1p, *Kl* Tar1p and 3HA-Tar1p were detected using a specific anti-Tar1p antibody. (A) Subcellular localization of endogenous Tar1p and tagged 3HA-Tar1p in *S. cerevisiae* W303 strain. The 3HA-Tar1p is expressed from a high copy vector. Mitochondria samples (M20, 20 µg or M100, 100 µg), T samples (5 µg) and PM samples (20 µg) were not heated before loading. Endogenous *Sc* Tar1p is hardly detectable in 20 µg of mitochondrial extract. (B) Immuno-detection of *Sc* Tar1p is sensitive to heating. As indicated, mitochondria samples (M100, 100 µg or M200, 200 µg) extracted from W303 strain, were or not heated before loading. (C) Mitochondria from *K. lactis* contain a Tar1p-like protein. Mitochondria from *K. lactis* were purified following the protocol used for *S. cerevisiae*. Mitochondria from *S. pombe* (*Sp*) were obtained from N. Bonnefoy. Mitochondria samples (*Sc* 100 µg, *Kl* 20 µg and *Sp* 100 µg) were not heated before loading excepted when indicated. The protein Cox2p was detected with *Sc* or *Sp* anti-Cox2p antibody as indicated. Asterisks indicate the position of non-specific signals or putative oligomeric forms of *Sc* and *Kl* Tar1p polypeptides (see also Figure 3).

**Figure 3:**
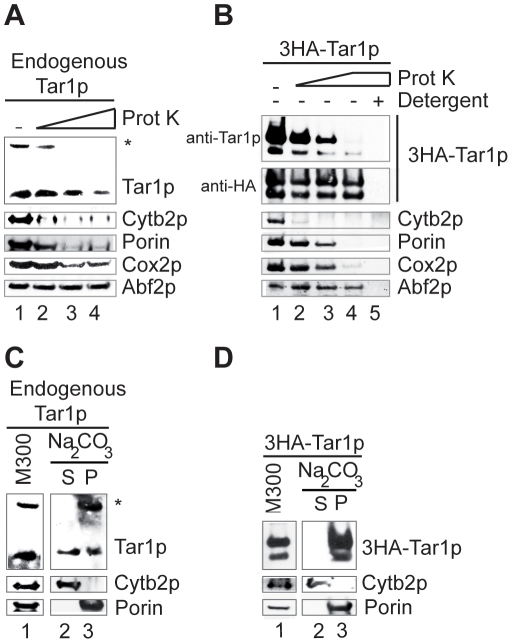
Endogenous Tar1p and tagged 3HA-Tar1p are associated with the mitochondrial inner membranes. (A, C) Mitochondria purified from W303 strain or (B, D) from W303 strain expressing tagged 3HA-Tar1p were (A, B) treated with proteinase K (Prot K) or (C, D) extracted with carbonate sodium (Na_2_CO_3_). Proteins were analyzed by immunoblotting with anti-cytochrome *b*_2_ as an intermembrane space protein marker (Cytb2p), anti-porin as an integral outer membrane marker (Porin), anti-Cox2p as an integral inner membrane marker (Cox2p) and anti-Abf2p as a matrix marker (Abf2p). Unless indicated, Tar1p and 3HA-Tar1p were detected with the anti-Tar1p antibody. (A) Mitochondria (100 µg) were treated with increased amount of proteinase K: 0.5, 1, and 4 µg (lanes 2–4). Lane 1: no treatment (-). (B) Mitochondria (20 µg) were treated with increased amount of proteinase K: 0.5, 1, and 4 µg (lanes 2–4). Lane 1: no treatment (-), lane 5: proteinase K (4 µg) treatment in the presence of 1% Triton (Detergent, +). (C, D) Mitochondria (M300, 300 µg, lane 1) were incubated with Na_2_CO_3_ and separated into soluble supernatant (S, lane 2) and membrane pellet (P, lane 3) fractions by centrifugation. Porin was used as an integral membrane marker and Cytb2p as a soluble protein.

To assess the specificity of the anti-Tar1p antibody, we first used it to detect a version of *Sc* Tar1p tagged with 3 copies of the haemagglutinin (HA) epitope at the amino-terminal end (3HA-Tar1p) and expressed from a plasmid in the wild-type background W303 (Figure 2A). The 3HA-Tar1p polypeptide consists of three copies of the HA epitope fused to *TAR1* ORF and has a predicted size of 19.7 kD. Considering that *Sc TAR1* ORF tagged at the 3′ end was reported to be located in mitochondria [8], we assayed 3HA-Tar1p immuno-detection in whole cell extract, postmitochondrial supernatant and purified mitochondria prepared from the 3HA-Tar1p containing strain. One immunoreactive species with an apparent molecular weight of 25 kD was detectable in whole cell extract (Figure 2A, lane 5) and an additional band of 21 kD was detected in purified mitochondria, suggesting that the 3HA-Tar1p polypeptide undergoes a proteolytic cleavage (Figure 2A, lane 7). In comparison, no bands were recognized by the anti-Tar1p antibody in the postmitochondrial supernatant (Figure 2A, lane 6) or in the whole cell lysate and postmitochondrial supernatant from strain W303 carrying an empty plasmid (Figure 2A, lanes 1–2). Meanwhile, probing with an anti-HA antibody revealed the same immunoreactive species as those detected with the anti-Tar1p antibody (Figure 3B and data not shown). All together, these results established the specificity of our anti-Tar1p antibody.

Then, detection of the endogenous *Sc* Tar1p was assayed in whole cell lysates and purified mitochondria from strain W303. Whereas we did not detect the endogenous protein in total cell extracts (Figure 2A, lane 1), we did detect *Sc* Tar1p in purified mitochondria (Figure 2A, lane 4). The anti-Tar1p antibody recognizes one mitochondrial immunoreactive species with an apparent molecular weight of 12 kD that is compatible with the expected size of endogenous Tar1p (14.3 kD). Importantly, the immuno-detection required that at least 100 µg of purified mitochondria were loaded on the gel (Figure 2A, lanes 3–4) and the signal was found strongly reduced when mitochondrial extracts were heated before loading (Figure 2B, compare lanes 3–4 to 1–2). Such thermo-labile property was not observed for the mitochondrial matrix marker Abf2p (Figure 2B) or for the mitochondrial inner membrane marker Cox2p (Figure 2A). Bonawitz and colleagues have previously reported that endogenous *Sc* Tar1p was neither detectable in whole cell extracts nor in purified mitochondria [10]. Since we have unambiguously detected Tar1p in mitochondria, we suspect that the authors used either heated samples, too low amount of purified mitochondria or had low quality antibodies.

The presence of *TAR1*-like ORF nested antisense the rDNA was shown conserved in several hemiascomycetous yeasts [8] with the length of the predicted Tar1p-like proteins varying from 64 to 124 residues (see examples in Figure 1B). The predicted Tar1p-like protein of the yeast *K. lactis* is 109 aas in length, it shares 78.2% sequence identity with *Sc* Tar1p and sequence of the antigenic peptide TKNRTPRHTGFSPS is well conserved (Figure 1B). We thus asked whether an endogenous *Kl* Tar1p polypeptide could be detected using *S. cerevisiae* anti-Tar1p antibody. Mitochondria from *K. lactis* wild type strain CBS2359 were isolated following the protocol used for *S. cerevisiae* (see Materials and Methods). Three immunoreactive bands were detected in *K. lactis* purified mitochondria, the major one having the higher mobility and the same apparent molecular weight (12 kD) as *Sc* Tar1p endogenous protein (Figure 2C, lanes 1–2). The two bands of slower mobility had apparent molecular weights of 45 kD and 29 kD. A similar 29 kD immnunoreactive signal can be detected in *S. cerevisiae* mitochondrial extracts (Figure 2C, lane 1; see also Figure 2B). As observed for *Sc* Tar1p, the *Kl* Tar1p immunoreactive signals appeared thermo-labile (Figure 2C, compare lanes 2 and 3). This heating-sensitivity was not observed for *K. lactis* Cox2p detected on the same western blot using *S. cerevisiae* anti-Cox2p antibody. In contrast to *Sc* Tar1p, *Kl* Tar1p can be detected in 20 µg of purified mitochondria suggesting that the endogenous *K. lactis* polypeptide is more stable or more expressed than the *S. cerevisiae* protein. The 29 kD and 45 kD detected bands have molecular mass that could correspond to oligomeric forms of the Tar1p polypeptides but these signals could as well being non-specific.

Next, we tested whether the mitochondria from the fission yeast *Schizosaccharomyces pombe* could contain a Tar1p-like product although we did not detect the presence of a *TAR1*-like ORF in the rDNA units or in the whole genome of this organism. No specific bands were recognized by *S. cerevisiae* anti-Tar1p antibody in *S. pombe* purified mitochondria (Figure 2C, lane 4). Meanwhile, the mitochondrial inner membrane marker Cox2p was efficiently detected in these *S. pombe* mitochondrial extracts.

Thus, the product of the rDNA-nested antisense gene *TAR1* can be detected as an authentic mitochondrial polypeptide in two hemiascomycetous species. *Sc* and *Kl* Tar1p endogenous polypeptides show the same apparent molecular weight although their predicted lengths differ from fifteen residues. The first methionine residue of the *Kl* protein-sequence corresponds to the second methionine residue of *Sc* protein-sequence and both sequences contain an additional proximal methionine residue (see Figure 1B). This suggests that in *S. cerevisiae* the used initiator codon might be the second (or third) in-frame ATG codon or alternatively, that the N-terminal end of *Sc* Tar1p is cleaved (see below and Discussion).

### The endogenous *S. cerevisiae* Tar1p is associated with mitochondrial inner membranes

The detection of endogenous Tar1p prompted us to determine its submitochondrial location in the yeast *S. cerevisiae*. We first examined Tar1p sensitivity to digestion by exogenous proteinase K added to purified mitochondria. This protease treatment degraded the outer membrane marker protein porin and the intermembrane space marker protein cytochrome *b2* (Cyt b_2_) indicating that the outer membrane of purified mitochondria was a little damaged (Figure 3A). Nevertheless, the matrix marker protein Abf2p was protected from digestion. In comparison to porin and Cyt b_2_, endogenous Tar1p was more resistant to protease treatment, a behavior similar to that of the inner membrane marker protein Cox2p (Figure 3A).

We performed the same experiment to determine the protease sensitivity of the tagged protein 3HA-Tar1p. In this case, the western blot was successively probed with the anti-HA and the anti-Tar1p antibodies. Tagged protein 3HA-Tar1p appeared highly resistant to digestion by proteinase K when detected with the anti-HA antibody, which recognizes the N-terminal 3HA epitopes (Figure 3B). In comparison, probing with the anti-Tar1p antibody, which recognizes a C-terminal epitope (residue 79 to 92; Figure 1B), revealed a sensitivity of 3HA-Tar1p towards digestion and a behavior similar to that of the endogenous Tar1p (Figure 3A and 3B, lanes 2–4). For each probing condition (anti-HA and anti-Tar1p), the 3HA-Tar1p bands of 25 kD and 21 kD showed the same behavior towards the protease treatment. Detergent solubilization of the membranes rendered 3HA-Tar1p entirely sensitive to protease (Figure 3B, lane 5). The difference in resistant profiles indicates that the N-terminal part of the tagged protein 3HA-Tar1p is protected from the protease digestion (as is the matrix marker protein Abf2p) whereas the C-terminal end is exposed.

Hydropathy analysis suggests that Tar1p contained one putative membrane spanning-segment (residues 21 to 41; Figure 1B–C). We thus tested endogenous Tar1p and tagged protein 3HA-Tar1p for membrane association (Figure 3C–D). Purified mitochondria were treated with sodium carbonate that disrupts all mitochondrial membranes. Afterwards, soluble and membrane fractions were separated by ultracentrifugation, subjected to SDS-electrophoresis and probed with anti-Tar1p antibody. Endogenous Tar1p was mainly recovered in the membrane fraction and to a less extent in the soluble fraction, indicating that a large amount of the protein could not be extracted from mitochondrial membranes (Figure 3C). Also, endogenous Tar1p is mainly associated with membranes. The 25 kD and 21 kD immunoreactive species of the tagged protein 3HA-Tar1p could not be extracted from mitochondrial membranes, indicating that they are also integral membrane polypeptides (Figure 3D).

In summary, carbonate treatment shows that endogenous and tagged proteins Tar1p are associated with mitochondrial membranes. Protease treatment indicates that polypeptides reside in the mitochondrial inner membranes most probably in a N_in_-C_out_ topology because the C-terminus of the endogenous and tagged protein is exposed to protease digestion whereas the N-terminus of the tagged protein is not. These results are fully consistent with the physical interaction detected between Tar1p and the methyltransferase Coq5p [10], which is a mitochondrial matrix protein peripherally associated with the inner membrane [14].

### Neither the N-terminus nor the C-terminus of *S. cerevisiae* Tar1p are critical for mitochondrial localization

Compared to the amino-acids sequence of *S. cerevisiae* Tar1p, Tar1p-like sequences can be truncated at their N-terminal end, their C-terminal end or both (see examples in Figure 1B). As mentioned above, the amino-acids sequence of *Kl* Tar1p does not possess the first fifteen residues of *Sc* Tar1p sequence, nevertheless endogenous *K. lactis* protein does localize to mitochondria (Figure 2C).

**Figure 4:**
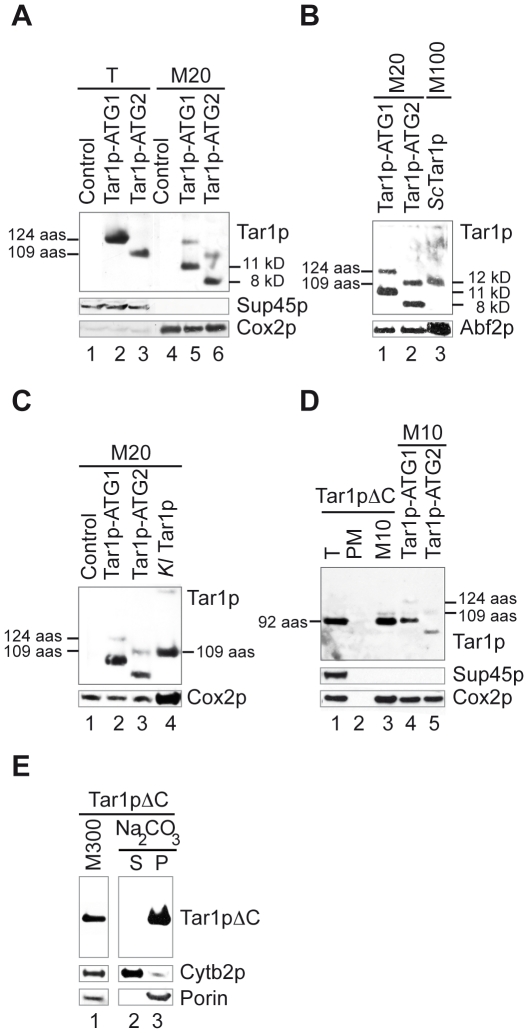
*Sc* Tar1p polypeptide truncated at its N-terminus or C-terminus co-fractionates with mitochondria. (A) Total cell extracts (T, 5 µg) and mitochondrial extracts (M20, 20 µg) from W303 strain expressing an empty plasmid (control) or plasmid-version of Tar1p (Tar1p-ATG1, 124 aas) or of N-terminal truncated Tar1p (Tar1p-ATG2, 109 aas). The amino-acids length of plasmid-derived polypeptides is indicated. The apparent molecular weight of smaller immunoreactive species detected in mitochondrial extracts is indicated. (B, C) Compared electrophoretic mobility between endogenous *Sc* Tar1p, endogenous *Kl* Tar1p and plasmid-derived Tar1p-ATG1 and Tar1p-ATG2 in mitochondrial extracts. Mitochondrial extracts are the same as in (A) and from *S. cerevisiae* and *K. lactis* wild type strains (see Figure 2). (D) Total cell extracts (T, 10 µg), postmitochondrial supernatant (PM, 10 µg) and mitochondria (M10, 10 µg) from W303 strain expressing a plasmid-derived C-terminal truncated Tar1p (Tar1pΔC, 92 aas). Mitochondria (M10, 10 µg) from W303 strain expressing Tar1p-ATG1 or Tar1p-ATG2 were loaded in parallel to compare the apparent molecular weight of the different immnuoreactive species. Lane 3, the thin band detected above the Tar1pΔC signal may correspond to endogenous *Sc* Tar1p. (E) Mitochondria (M300, 300 µg) from W303 strain expressing Tar1pΔC were incubated with Na_2_CO_3_ and separated into soluble supernatant (S) and membrane pellet (P) fractions. The different Tar1p polypeptides were revealed using the specific anti-Tar1p antibody.

To address whether the first fifteen residues of *Sc* Tar1p participated in the mitochondrial localization of the *S. cerevisiae* protein, we constructed two non-tagged plasmid-versions of *TAR1* ORF: one encoding a 124 aas polypeptide that starts at the first ATG codon (Tar1p-ATG1) and one encoding a 109 aas polypeptide that starts at the second ATG codon (Tar1p-ATG2). Both constructions were expressed under the control of the promoter of the *PGK1* gene on a high copy plasmid. We took advantage of the non-detection of the endogenous *Sc* Tar1p protein within low amounts of mitochondria to detect the plasmid-derived polypeptides in 20 µg of mitochondrial extracts from strain W303 expressing plasmids. Whereas unique immunoreactive species of the expected size were detected in whole cell extracts (Figure 4A, lanes 2–3), two bands were revealed in mitochondria (Figure 4A, lanes 5–6) suggesting that plasmid-derived Tar1p-ATG1 and Tar1p-ATG2 polypeptides underwent a proteolytic cleavage. In both cases, the smaller immunoreactive species were more abundant than full-length polypeptides and their apparent molecular weights suggest they were about thirty residues shorter. Since both Tar1p-ATG1 and Tar1p-ATG2 polypeptides similarly localize to mitochondria, the N-terminal end (residues 1 to 15) of *Sc* Tar1p was found dispensable for targeting.

As shown in Figure 4B and 4C, both *Sc* and *Kl* endogenous Tar1p migrated at the same apparent molecular weight as the full-length plasmid-derived Tar1p-ATG2 polypeptide (109 aas). While expected for *Kl* Tar1p, this result indicates that mitochondrial *Sc* Tar1p is probably also 109 aas long suggesting once again that the initiator codon is the second in-frame ATG codon or that N-terminal end of endogenous Tar1p is cleaved. The latter hypothesis seems however less probable because the plasmid-derived Tar1p-ATG1 polypeptide was not matured to a 109 aas product but to a shorter one (Figure 3 B).

Next, we constructed a plasmid-version of the *TAR1* ORF encoding a polypeptide shortened of 32 residues at its C-terminal end (Tar1pΔC, 92 aas). The construction uses the first ATG codon as initiator codon and ends immediately after the epitope specifically recognized by the anti-Tar1p antibody (see Figure 1B). The 3′ end truncated ORF was expressed under the control of *PGK1* promoter on a high copy plasmid. The Tar1pΔC product fractionated with mitochondria as one immunoreactive species, which had the same apparent molecular weight as the unique signal detected in whole cell extract (Figure 4D, lanes 1 and 3). Also, in this case, a proteolyzed form was not detected in purified mitochondria, indicating that the C-terminal truncated polypeptide became resistant to cleavage. Finally, the Tar1pΔC protein was found completely resistant to extraction by carbonate showing that it was strongly embedded in the mitochondrial membranes (Figure 4E).

These results show that neither the N- nor the C-terminal ends of *Sc* Tar1p are required for protein targeting to mitochondria. Moreover, Tar1p truncated of its last 32 aas is still associated with mitochondrial membranes. Altogether, it indicates that Tar1p is generated without a presequence and its targeting might depend on internal segment(s) (see Discussion). We noticed that with the exception of the Tar1pΔC construct, all plasmid-derived Tar1p polypeptides (3HA-Tar1p, Tar1p-ATG1 and Tar1p-ATG2) gave rise to a second immunoreactive species in mitochondrial extracts that, in each case, had an apparent size shortened of about thirty residues. In addition, immunoreactive species derived from the N-terminal tagged 3HA-Tar1p can be revealed with anti-HA antibody (Figure 2A and 3B). Thus, we suspect that a proteolytic cleavage site exists within the last 32 residues of *Sc* Tar1p amino-acid sequence. However, such maturation event was not observed for *S. cerevisiae* or *K. lactis* endogenous proteins.

### *TAR1* transcripts are low abundant and display extensive 5′ and 3′ heterogeneity

High frequency with which the short *S*. *cerevisiae TAR1* ORF has been identified in the gene-trapping approach, led to the proposal that many, if not all *TAR1* copies in the rDNA were transcribed [6] [8]. This transposon-insertion approach has been carried out into the genetic background Y800 and for strains grown in glucose medium [6]. Using northern blot analyses, we hardly detected *TAR1* transcripts in mRNAs purified from wild-type strain W303 grown in glucose or galactose rich medium (data not shown). Also, *TAR1* transcripts might be rather weakly expressed or unstable hence, the difficulty to detect endogenous Tar1p protein.

**Figure 5:**
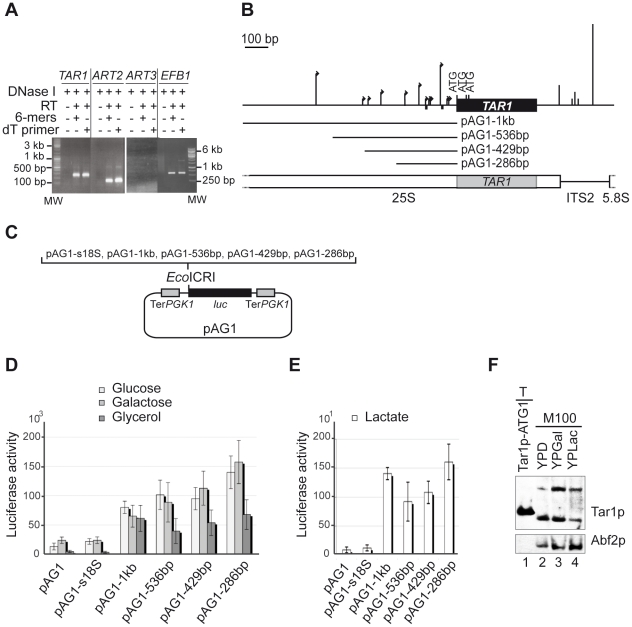
Characterization of the *TAR1* transcripts - Expression of *TAR1* in fermentable and non-fermentable conditions. (A) RT-PCR analyses were performed on total RNA extracted from wild-type W303 strain grown in galactose medium. After DNase treatment (DNase I, +) and reverse transcription (RT, +) with random hexamers (6-mers, +) or oligodT primer (dT primer, +), PCR was performed using gene-specific primers (as indicated; Table S1). Samples without RT (-) were used as controls for DNA contamination. RT-PCR product's length (600 bp) generated from the transcript of the intron-containing gene *EBF1* confirmed the absence of genomic contamination. Expecting sizes of RT-PCR products were *TAR1*: 258 bp, *ART2*: 156 bp, and *ART3*: 204 bp. (B) Schematic representation of *TAR1* showing 5′ and 3′ ends mapped by 5′ and 3′ RACE. Size of arrows (5′ ends) and of vertical lines (3′ ends) is proportional to the number of amplification products identified at indicated positions: −37 (x1), -45 (x1), −75 (x6), −123 (x1), −130 (x1), −146 (x1), −178 (x3), −241 (x2), −355 (x2), −415 (x1), −440 (x1), and −657 (x5) (5′ ends); +109 (x3), +170 (x1), +181 (x2), +198 (x1), and +266 (x12) (3′ ends). Numbering refers to −1 as the first residue upstream the first ATG codon and to +1 as the first residue beyond the TGA stop codon. Small black squares represent putative TATA elements (−73 and −144). ITS2: internal transcribed sequence 2 of Pol I transcript. Grey box represents *TAR1* ORF in the 25S rDNA. *TAR1* 5′ flanking regions tested in (C) are indicated (pAG1-1 kb, pAG1-536 bp pAG1-429 bp, pAG1-286 bp). (C) Schematic representation of the plasmid-borne reporter system used to test promoter activity of *TAR1* 5′ flanking regions. The empty vector pAG1 and the pAG1-s18S construct were used as negative controls. pAG1-s18S contains a 500 bp region of 18S rDNA devoid of small ORFs. Ter*PGK1*: terminator of the *PGK1* gene; *luc*: Firefly luciferase gene; *Eco*ICR1: cloning site. (D–E) Histograms showing luciferase activities from indicated construct and indicated growth condition. The values (in relative light units per milligram of total protein per second) are averages of five independent assays. Error bars are indicated. Note the different scales of the two histograms. (F) Expression of endogenous Tar1p in fermentable or non-fermentable carbon sources. Mitochondria (M100, 100 µg) were purified from W303 strain grown in glucose (YPD), galactose (YPGal) or lactate (YPLac) rich medium. Tar1p and the matrix marker Abf2p were detected with the anti-Tar1p and anti-Abf2p antibodies, respectively. Five µg of total cell extract (T) from W303 strain expressing plasmid-borne Tar1p-ATG1 (124 aas) were loaded in parallel.

RT-PCR experiments were previously used to detect the endogenous *TAR1* mRNA in total RNA purified from strain Y800 grown at stationary phase [10]. Here, we performed RT-PCR on total RNA samples isolated from strain W303 grown to mid-log phase in glucose or galactose rich medium. In parallel, we also assayed detection of the transcripts of *ART2* and *ART3*, the two other ORF nested antisense the rDNA (Figure 1A). In both fermentable growth conditions, a RT-PCR product of the expected length was generated from *TAR1* and *ART2* mRNAs whereas none was amplified from the *ART3* transcript (Figure 5A and data not shown). Equivalent results were obtained when reverse transcription was carried out using a random or an oligo dT primer and no PCR product was amplified in the absence of RT.

Next, we used rapid amplification of cDNA ends (RACE) to characterize the 5′ and 3′ ends of endogenous *TAR1* transcript (see Materials and Methods for details). Amplification products were cloned and sequenced individually to map 5′ and 3′ cDNA ends. Nucleotide sequences identified multiple 5′ and 3′ ends indicative of alternate promoter and terminator uses (Figure 5B). No fewer than twelve different 5′ ends were identified, located 37–657 bp upstream the first ATG codon of *TAR1* ORF. The more frequently identified 5′ ends are 657 and 75 bp upstream the *TAR1* ORF but only the proximal one is very closed to a TATAA element (−73 bp; Figure 5B). Five different 3′ ends were identified, located 109–266 bp beyond *TAR1* stop codon. The major 3′ end falls in an A-rich element located 266 bp beyond *TAR1* stop codon (Figure 5B).

In conclusion, endogenous *TAR1* transcripts are present at low level, which is in agreement with the non-detection of Tar1p in crude cell extracts and the need for large amounts of purified mitochondria to detect it. The substantial 5′ and 3′ heterogeneity of *TAR1* transcripts may result from the atypical genomic location of the *TAR1* gene within rDNA repeats (see Discussion).

### *TAR1* is expressed in fermentable and non-fermentable growth conditions

We quantified the promoter activity of the *TAR1* gene using a low-copy plasmid-borne reporter system in which the expression of the firefly *luc* gene was driven by either *TAR1* 5′ flanking sequences (286 bp to 1 kb; Figure 5B–C), the promoter of the *PGK1* gene (used as a positive control), or a 500 bp fragment of 18S rDNA (pAG1-s18S construct used as a negative control). Luciferase activity was measured for W303 cells grown to mid-log phase in fermentable (glucose, galactose; Figure 5D) or non-fermentable (glycerol, lactate; Figure 5D–E) medium. During non-fermentable and fermentable growth, *PGK1* promoter gave rise to activities 600 to 1400-fold higher than control constructs, respectively (controls were empty vector pAG1 or pAG1-s18S vector; see Figure 5C and data not shown). In comparison, tested *TAR1* 5′ flanking sequences showed a modest but significant (p-values ≤0.024) promoter activity 3 to 7-fold higher than controls when W303 strains used glucose or galactose as carbon sources (Figure 5D). Equivalent *TAR1* promoter activities were quantified in glucose and galactose medium and in both cases shortening of *TAR1* 5′ flanking sequence to 286 bp (pAG1-286 bp construct) significantly enhanced expression of the reporter *luc* gene when compared to the pAG1-1 kb construct (Figure 5D; p-values  = 0.008). In contrast, when strains were grown in non-fermentable carbon sources, shortening of *TAR1* 5′ flanking sequences did not significantly change luciferase activity (Figure 5D–E). It could suggest that negative regulatory elements responsive to fermentable growth conditions were present in distal position of the *TAR1* 5′ flanking region.

For all plasmid constructions, our reporter system gave rise to luciferase activities of lesser magnitude when strains were grown in non-fermentable (glycerol, lactate) compared to fermentable medium (glucose, galactose; Figure 5 D–E). Luciferase expression driven by the *TAR1* 5′ flanking regions was however 7 to 14-fold higher than controls indicating that their enhancer effect was more important in respiratory than in non-respiratory growth. This is consistent with the reported induced expression of a chromosomal *TAR1*-lacZ fusion in glycerol *versus* glucose medium [10]. Using western blot analyses, we found that the endogenous Tar1p protein was however less detectable in growing condition that requires respiration (lactate), than in glucose or galactose (Figure 5F), Thus, whereas reporter systems [10] (and this work) indicated an induction of *TAR1* expression under respiratory conditions, our western analyses did not show a correlated increase in the amount of endogenous Tar1p. This apparent discrepancy could be simply explained by the instability of endogenous Tar1p in lactate medium or by a different turn over of the Tar1p, luciferase (This work) and β-galactosidase [10] polypeptides.

## Discussion

In the present work, we demonstrate for the first time that the *TAR1* gene nested antisense to the nuclear rDNA repeats, encodes an authentic protein, which localizes to the mitochondria from *S. cerevisiae* and *K. lactis* hemiascomycetous yeasts. Detailed localization of *S. cerevisiae* Tar1p further indicates that Tar1p is associated with the mitochondrial inner membrane.

Mitochondrial *Sc* and *Kl* Tar1p share the same apparent molecular weight (12 kD) although *Sc* Tar1p is predicted to be fifteen residues longer at its N-terminal end. It may suggest that the N-terminal end of *Sc* Tar1p protein was cleaved off in mitochondria. An alternative is that *Sc* Tar1p was generated by initiation of translation at the second (or third) in-frame AUG codon although according to the scanning model for translation, the first AUG codon is ordinary preferred. We examined the nucleotide context of the three in-frame AUG codons of *Sc TAR1* ORF. In yeast, the preferred consensus sequence is 5′-(A/Y)A(A/U)A AUG UCU-3′ with the A in position -3 being the most highly conserved residue surrounding the AUG codon [15] [16]. Only the second AUG codon of *Sc TAR1* ORF has the appropriate A residue in this position whereas the first and third AUG contain a residue G or C at this site, respectively. In addition, the second AUG is followed by the prevailing UCU serine codon whereas the first and third AUG codons are followed by rare CGA and CCC codons, respectively. Thus, the sequence context of the second AUG is more favorable than the one of the first AUG, a situation that might promote translation from the second start codon.

**Figure 6:**
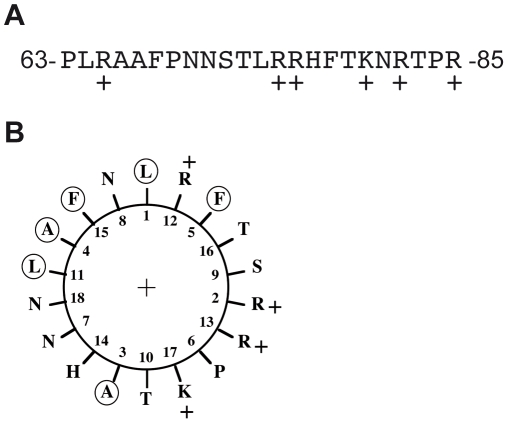
The positively charged region of *Sc* Tar1p. (A) The internal 23 amino acids of Tar1p with the positive residues, +. (B) α-helical plot of 18 amino acids (residues 64 to 81) constructed using the DNA strider program [40]. Apolar residues are circled.

Since endogenous *Sc* Tar1p protein could not be detected in whole cell extract, we cannot settle between these two hypotheses (cleavage of the N-terminal extension or translation initiation). We can however exclude that the *Sc* N-terminal extension (if present) acts as a mitochondrial targeting sequence since: (i) it is not predicted to form an amphipathic α-helix and moreover contains negatively charged residues [17]; (ii) its removal does not prevent mitochondrial localization; (iii) the addition of three HA epitopes at the N-terminus of *Sc* Tar1p does not either prevent mitochondrial localization; (iv) *Kl* Tar1p devoid of N-terminal 15 aas extension localizes to mitochondria. As many mitochondrial inner-membrane proteins [18], Tar1p is thus generated without an amino-terminal presequence. Numerous other types of targeting signals, which are located at various positions within mitochondrial proteins, have been described [19] (for review). We noticed that Tar1p primary sequences contain a stretch of positively charged amino acid residues after the hydrophobic region of the proteins (Figure 6A; see also Figure 1B). Such composite sequence arrangement can constitute an internal mitochondrial-targeting signal for proteins associated with the inner membrane [19]. For example, this has been demonstrated for yeast Bcs1p, cytochrome c_1_ and Tim23p that are anchored in the inner membrane *via* one (Bcs1p, cytochrome c_1_) or four (Tim23p) transmembrane domains [20] [21] [22]. Whereas Bcs1p and Tim23p do not carry an amino-terminal presequence, cytochrome c_1_ has one that operates independently from the internal signal [21]. It was proposed that the positively charged segment of these proteins has the potential to form an amphipathic α-helix, like presequences. The internal 18 aas segment (residues 64 to 81) of *Sc* Tar1p similarly displays the ability to form an amphipathic α-helix (Figure 6B). The corresponding segment of *Kl* Tar1 has the same ability (data not shown).

Targeting of *Sc* Tar1p to mitochondrial inner membranes is preserved even if the last 32 residues of the polypeptide are deleted. As illustrated in Figure 1B, some Tar1p-like polypeptides do not contain the C-terminal end present in *S. cerevisiae* and *K. lactis* sequences and these shorter Tar1p amino acid sequences end just after the positively charged region [8]. Also, the conserved core of Tar1p polypeptides is made up of a putative transmembrane domain (TM, Figure 1B–C) and of a targeting signal resembling those of hydrophobic inner membrane associated proteins [19]. We found that the C-terminal end of plasmid-derived *Sc* Tar1p polypeptides probably undergoes a proteolytic processing event in mitochondrial extracts. Such processing was not observed for the low abundant endogenous protein suggesting that it could be triggered by high expression of Tar1p polypeptides, which would become targets of protease(s). Additional studies will be required to determine whether such proteolytic event might participate in Tar1p regulation.

Detection of the endogenous *TAR1* product definitively ascertains that the *TAR1* gene forms a transcription unit within rDNA repeats. Whereas the rDNA region is highly transcribed by RNA polymerases (Pol) I and III, it is known that Pol II-transcribed genes integrated into the rDNA are silenced [3] [4]. However, chromatin immunoprecipitation (ChIP) analyses showed two sites of pol II occupancy in the yeast rDNA, one at a characterized bidirectional E-pro promoter and one in the vicinity of the *TAR1* gene [5]. The Pol II E-pro promoter was identified in the intergenic spacer region, which separates two rDNA repeats [23]. The two divergent non-coding transcripts generated from the E-pro promoter were proposed to regulate rDNA copy number and rDNA stability [4] [24] [25]. Both E-pro and *TAR1*-close promoters showed a Pol II enrichment dependent of the Sen1p helicase, which is a Pol II termination factor for short non coding and protein-coding genes in yeast [5]. Sen1p works in complex with the RNA-binding proteins Nrd1p and Nab3p, which can be targeted to transcripts carrying the recognition sequences GUA(A/G) and UCUU, respectively [26] [27] [28]. Whereas the longest observed *TAR1* transcript contains multiple potential binding sites for Nab3p, there are only two potential binding sites for Nrd1p, one in the 5′ end and the other in the 3′ end. Nrd1p binds to early elongating-Pol II enzyme thereby targeting the Nrd1-Nab3-Sen1 complex to 5′ regions of genes and promoting Sen1p-dependent termination pathway [28]. Nrd1p also associates to the 3′ to 5′ exosome thus influencing RNA degradation [29] [30]. Also, we can speculate that the Nrd1-Nab3-Sen1 complex may participate in the regulation of *TAR1* expression. In addition, 3′ end heterogeneity of *TAR1* transcripts may also result from the collision with the oncoming Pol I molecules as previously proposed for the antisense IGS1-R transcripts generated from the E-pro promoter [25].

To date, the function of Tar1p remains unknown. The localization of *S. cerevisiae* endogenous protein in association with the mitochondrial inner membrane is consistent with the two-hybrid interaction reported with the Coq5p protein [10], which is peripherally associated with the inner mitochondrial membrane on the matrix side [14]. Along with nine other yeast genes, *COQ5* was shown to be required for the endogenous biosynthesis of the coenzyme Q, a critical component of the electron transport pathways [31] (for review). However, the putative role of Tar1p in the biosynthesis of coenzyme Q remains to be clarified. Tar1p function might be not restricted to hemiascomycetous yeasts. We looked for *S. cerevisiae* Tar1p homologs by performing BLASTp searches (BLASTP 2.2.23, May 2010) and detected candidates beside the Saccharomycotina subphylum, for example, one hypothetical protein of the platyhelminthe *Schistosoma japonicum* (99 aas in length; accession ABA40770.1) and one of the archamoeba *Entamoeba histolytica* (111 aas in length; accession XP_001914542.1). In both cases, the *TAR1*-like genes were similarly nested antisense to 25S rDNA sequences. The hypothetical polypeptides share 30.6% and 34.7% identity with the *Sc* Tar1p sequence, respectively and they possess the conserved core of Tar1p polypeptides within which sequence identity with *Sc* Tar1p reaches 54%.

To comprehend the role, if any, of the antisense *TAR1* gene, further investigations will be required. Classical functional analyses by inactivation or deletion of this gene are obviously challenging due to its genetic location within highly constrained and repeated rDNA sequences. However, it was recently reported that artificial box C/D RNA can be successfully used to specifically guide mRNA modification thus interfering with gene expression [32] [33]. This new molecular approach certainly represents an opportunity to attempt inactivation of *TAR1* gene expression *via* the 2′-O-methylation targeting of its transcripts.

## Materials and Methods

### Yeast strains and growth conditions

The *Saccharomyces cerevisiae* strain used in this study is W303 (*Matα ade2-1*, *trp1-1*, *ura3-1*, *his3-11,15*, *leu2-3*). The *Kluyveromyces lactis* strain is wild-type CBS2359 (*MAT* ATCC8585). For mitochondria isolation, unless otherwise indicated, W303 strain and derivatives were grown in 2% galactose-0,1% glucose rich medium plus adenine (20 mg/L) or in 2% galactose-0,1% glucose complemented selective medium (CSM, BIO-101) plus adenine (20 mg/L) for strains containing plasmids. Cells were collected at OD_600 nm_ = 2. Luciferase activities were measured for *S. cerevisiae* strains grown in either 2% glucose, 2% galactose, 2% glycerol or 0,5% lactate CSM medium, as indicated. In this case, cultures were harvested at OD_600 nm_ = 1.5. For RNA isolation, W303 strain was grown in 2% galactose-0,1% glucose and cells collected at OD_600 nm_ = 1.

### Plasmid-derived Tar1p polypeptides

For all constructs, *TAR1* sequences were amplified by PCR from an rDNA sequence borne on vector pFL44L [34]. Constructs were confirmed by sequencing. Plasmid version of *TAR1* ORF tagged at its 5′ end with 3 copies of the haemagglutinin (HA) epitope was constructed as followed. The *TAR1* ORF was PCR amplified with the primer pair (5′*Eco*RI-TAR1) and (3′*Eco*RI-TAR1) (for all primers used in this study, see Table S1). The resulting PCR fragment contains the whole coding sequence except the first ATG codon. The PCR fragment was digested with *Eco*RI and cloned at the corresponding site in the high copy plasmid BFG1 (2 µm, *LEU2*; a gift of J. Camonis) that contains three copies of the HA epitope. The resulting BFG1-*TAR1* plasmid carries a 3HA-N-terminal tagged version of Tar1p (3HA-Tar1p) under the control of the promoter of the *PGK1* gene. Plasmid versions of full length and truncated *TAR1* ORF were constructed as followed. Full length *TAR1* ORF was amplified using the primer pair (5′*Bam*HI-TAR1) and (3′*Bam*HI-TAR1). The PCR product was digested with *Bam*HI and inserted into the *Bgl*II site of the high copy plasmid pEMBLye30/2 (2 µm, *LEU2*) [35]. The resulting pEMBL-*TAR1-*ATG1 plasmid carries the entire *TAR1* ORF (375 bp; polypeptide Tar1p-ATG1) under the control of the *PGK1* promoter. The pEMBL-*TAR1-*ATG2 plasmid that carries a *TAR1* ORF starting from the second ATG codon (330 bp; polypeptide Tar1p-ATG2), was similarly constructed using the primer pair (5′*Bam*HI-2ndTAR1) and (3′*Bam*HI-TAR1). The pEMBL-*TAR1-*ΔCter plasmid carrying a *TAR1* ORF truncated of its last 32 codons (279 bp; polypeptide Tar1pΔC) was constructed on the same vector using the primer pair (5′*Bam*HI-TAR1) and (3′*Bam*HI-TAR1-M93stop).

### Luciferase reporter-system

Constructions were done in the low copy vector pFL38 (*CEN URA3*) [34] as followed. First, terminator region (487 bp) of the *PGK1* gene was amplified by PCR from yeast genomic DNA using the primer pair (5′TerPGK1) and (3′TerPGK1). The PCR product was inserted into the *Pvu*II site and also into the *Sma*I site of pFL38. Second, The *luc* ORF (1653 bp) encoding luciferase enzyme was amplified by PCR from the plasmid p2Luc [36] using the primer pair (5′Firefly) and (3′Firefly). The PCR product was inserted into the *Eco*ICRI site of the vector between the two *PGK1* terminator sequences to yield plasmid pAG1, which conserves a unique *Eco*ICRI site downstream of the *luc* ORF (see Figure 5C). Third, 5′ flanking regions of the *TAR1* ORF were PCR-amplified from an rDNA sequence borne on vector pFL44L and inserted into the *EcoI*CRI site of pAG1. Primer pairs used to construct the pAG1-1 kb, pAG1-536 bp, pAG1-429 bp and pAG1-286 bp plasmids consist of the same 3′ primer (3′pTAR1) and the 5′ primers (5′1 kb-pTAR1), (pTAR1-536), (pTAR1-429) and (pTAR1-286), respectively. As a positive control, promoter of the *PGK1* gene (992 bp) was amplified by PCR using the primer pair (5′pPGK1) and (3′pPGK1) and inserted into the *EcoI*CRI site of pAG1. As a negative control, a rDNA fragment of 500 bp corresponding to the 3′ end of 18S sequence was amplified by PCR using the primer pair (500 pb-pAG1w) and (500 pb-pAG1c) and inserted into the *EcoI*CRI site of pAG1 yielding pAG1-s18S. All constructs were confirmed by sequencing.

### Luciferase assays

For each pAG1 construct, luciferase assays were performed on crude cell extracts (5 µl) from five transformants cultivated in the same conditions. Cells were broken in luc buffer (1% Triton X-100, 8 mM MgCl_2_, 1 mM DTT, 1 mM EDTA, 25 mM Tris-Phosphate pH 7.8, 15% glycerol) using the glass-beads method described [37]. Luciferase assays were performed in the presence of 2 µM ATP and 200 mM luciferin in luc buffer (100 µl). Light emission was measured during 10 seconds using a luminometer (Lumat LB9501). The protein concentration was determined for each cell extract using the method of Bradford (Bio-Rad Protein Assay). Luciferase activity was expressed as relative light units per milligram of protein per second.

### Miscellaneous

Mitochondria were isolated following classical differential-centrifugation procedures as described [38] with two modifications. Cells were resuspended in 1.2 M sorbitol buffer (1.2 M sorbitol, 50 mM Tris-HCl, pH 7.5, 10 mM EDTA, 0,3% 2-mercaptoethanol) at 3 ml/g wet mass cells before addition of zymolyase-100T (1 mg/g cells). The protein concentration was determined using the Bio-Rad assay. Protease digestion of mitochondria was carried out with 0.5, 1 or 4 µg proteinase K (Invitrogen). The mixtures were incubated for 20 min at 0°C and action of protease was halted by addition of 1 mM phenylmethylsulfonyl fluoride (PMSF) for 5 min on ice. When indicated, the reactions were carried out in the presence of 1% Triton X-100. For alkaline extraction with sodium carbonate, mitochondrial extracts were first reprecipitated at 17,000 g for 15 min. Pellets were resuspended in 100 mM Na_2_CO_3_ (pH 11.5), 5 mM DTT and protease inhibitors (Roche) and incubated on ice for 30 min. The membrane fraction was precipitated by centrifugation at 110,000 g for 1 hour (TL100; Beckman). The pellet was resuspended in 20 mM HEPES pH 7.4, 1 mM EDTA, 5 mM DTT with protease inhibitors. Supernatant and the pellet fractions were cleaned by centrifugation at 110,000 g for 30 min. Proteins of the supernatant were precipitated with 10% trichloroacetic acid (TCA) and resuspended in western blot loading buffer. Pellets were resuspended in loading buffer.

### Western blotting and antisera used in this study

Proteins were resolved on Pre-Cast gels (NuPAGE Bis-Tris gels, Invitrogen) and probed with the following antisera obtained from different sources. Anti-HA antibody (used at 1/5000, Eurogentec), *S. cerevisiae* anti-Cox2p (used at 1/500, Invitrogen), *S. pombe* anti-Cox2p (used at 1/5000, gift of N. Bonnefoy), anti-Cytb2p (used at 1/10000, gift of B. Guiard), anti-porin (used at 1/5000, Invitrogen), anti-Abf2p (used at 1/50000, C. Jacq, ENS, France), anti-Sup45p (used at 1/10000, gift of V. Heurgué-Hamard). Specific anti-Tar1p antibodies (used at 1/1000) were produced in an immunization program that included two successive immunizations and an affinity purification of rabbit polyclonal antibodies (Eurogentec).

### Transcripts analyses using RT-PCR and RACE PCR

Total RNA was isolated using the hot-phenol extraction method as described [39]. DNase treatment was performed using DNaseI RNase free enzyme (BioLabs). Reverse transcriptase reactions were carried out by standard procedures using Superscript II (Invitrogen) and random hexamers or oligodT primer. PCR amplifications were then independently performed from each cDNA sample using the following gene-specific primer pairs: *EBF1*: (ATG.EFB) and (TAA.EFB); *TAR1*: (5′*Bam*HI-TAR1) and (TAR1F78S); *ART2*: (5′ATG-ART2) and (3′ART2(156-137)); *ART3*: (5′ATG-ART3) and (3′ART3-TAA). The 5′/3′ RACE Kit, 2nd Generation (Roche) was used for amplification of 5′ and 3′ cDNA ends following manufacturer's instructions. For 5′ RACE reactions, cDNA amplification was carried out using the gene-specific primer (TAR1F78S). The PCR reactions successively used the primer pairs (antiTAR44-63)/(dT-anchor primer) and (antiTAR37-17)/(anchor primer). For 3′ RACE reactions, cDNA amplification was carried out using the (dT-anchor primer). The PCR reactions successively used the primer pairs (TAR*325-348)/(anchor primer) and (3′TAR1(350-375))/(anchor primer). Second amplification products were cloned in the vector pUC19 and sequenced individually.

## Supporting Information

Table S1 Primers used in this study. Primers are listed in the order they appear in the Materials and Methods section.
(DOC)
