# Supplementary material for: Mitochondria of the Yeasts Saccharomyces cerevisiae and Kluyveromyces lactis Contain Nuclear rDNA-Encoded Proteins
Source: PLoS One. 2011 Jan 25;6(1):e16325. doi: 10.1371/journal.pone.0016325 (PMC3026818; doi:10.1371/journal.pone.0016325)
Supplement: Table S1 — Primers used in this study. Primers are listed in the order they appear in the Materials and Methods section. (DOC) [file pone.0016325.s001.doc]

**Table S1.** Primers used in this study^a^

| Primer name | 5’-3’ sequence |
| --- | --- |
| (5’*Eco*RI-TAR1) | ccggaattccgagattcccctacccacaaggagc |
| (3’*Eco*RI-TAR1) | CCGGAATTCCGGTCAAATTTGAAATCTGGTACCTTCGG |
| (5’*Bam*HI-TAR1) | CGGGATCCCGATGCGAGATTCCCCTACCC |
| (3’*Bam*HI-TAR1) | cgggatcctcaaatttgaaatctggtaccttc |
| (5’*Bam*HI-2ndTAR1) | CGGGATCCCGATGTCTGATCAAATGCCCTTCCC |
| (3’*Bam*HI-TAR1-M93stop) | CGGGATCCTTAAGAGGGTGAGAATCCCGTGTGGCG |
| (5’TerPGK1) | CTCGAATTGAATTGAAATCGATAGATC |
| (3’TerPGK1) | CTCCCGATTGACCAATATATGTCTCTG |
| (5’Firefly) | GAGCTCACCATGGAAGACGCCAAAAACATAAAG |
| (3’Firefly) | TTACACGGCGATCTTTCCGCCCTTCTTGGCC |
| (3’pTAR1) | TTCACTGGGCCAGCATCAGTTTTGG |
| (5’1kb-pTAR1) | CCACTTCAGTCTTCAAAGTTCTC |
| (pTAR1-536) | ACCCCGGAACCTCTAATCATTC |
| (pTAR1-429) | TTTCGCCCCTATACCCAAATTC |
| (pTAR1-286) | ATCCGAAGACATCAGGATCG |
| (5’pPGK1) | CGCGAATTTTTCGAAGAAGTACC |
| (3’pPGK1) | TTATATTTGTTGTAAAAAGTAGATAATTACTTCC |
| (500pb-pAG1w) | TAATGATCCTTCCGCAGGTTCACC |
| (500pb-pAG1c) | TTTGTCTGCTTAATTGCGATAACG |
| (ATG.EFB) | ATGGCATCCACCGATTTCTC |
| (TAA.EFB) | TTATAATTTTTGCATAGCAG |
| (TAR1F78S) | GTGCGGTTCTTTGTAGAGTGCCTTCGAAGAG |
| (5’ATG-ART2) | ATGGTTTGTATTCACACTGAAAATC |
| (3’ART2(156-137)) | GAAGACATTGTCAGGTGGGG |
| (5’ATG-ART3) | ATGCCCCCTGGAATACCAAGGGG |
| (3’ART3-TAA) | TTATCTATTCATTAAATTTTTGTC |
| (antiTAR44-63) | GGGCATTTGATCAGACATGG |
| (dT-anchor primer) | GACCACGCGTATCGATGTCGACTTTTTTTTTTTTTTTT |
| (antiTAR37-17) | GTGCCCTCTGCTCCTTGTGGG |
| (anchor primer) | GACCACGCGTATCGATGTCGAC |
| (TAR*325-348) | CCCTCTCCAAATTACAACTCGGGC |
| (3’TAR1(350-375)) | CCGAAGGTACCAGATTTCAAATTTGA |

^a^Primers are given in the order they appear in the Materials and Methods section
